# Gross on Wounds of the Intestines and Artificial Anus

**Published:** 1843-08

**Authors:** 


GROSS ON WOUNDS OF THE INTESTINES AND ARTIFICIAL ANUS.

The paper on *Wounds of the Intestines*, published in the January, February, and March numbers of our last volume, and that on *Artificial Anus* contained in the Journal for July, have just been issued together, by Messrs. Prentice and Weissinger, in a volume of two hundred and twenty pages, octavo, with a colored lithograph illustrative of the first memoir. Our opinion of these articles has already been expressed. The first-named memoir is the result of about two years hard labor on the part of one who does things thoroughly. The memoir on Artificial Anus embodies a mass of information to be found nowhere else in the language. No volume of the same size, containing matter of a higher and more valuable character, has ever issued from the American press. C.

